# Relationship between Empowerment and Functioning and Disability in Older Japanese Patients: A Covariance Structure Analysis

**DOI:** 10.3390/healthcare11010044

**Published:** 2022-12-23

**Authors:** Yoshihito Tsubouchi, Akiyoshi Tainosho, Koudai Shimomura, Motoasa Kou, Kyosuke Yorozuya, Daiki Nakashima, Yasuo Naito

**Affiliations:** 1 Department of Rehabilitation, Naragakuen University, Nara-shi 631-8524, Japan; 2 Department of Rehabilitation, Akitsukounoike Hospital, Gose-shi 639-2273, Japan; 3 Department of Psychiatry, Akitsukounoike Hospital, Gose-shi 639-2273, Japan; 4 Faculty of Care and Rehabilitation, Seijoh University, Tokai-shi 476-8588, Japan; 5 Graduate School of Rehabilitation Science, Osaka Metropolitan University, Habikino-shi 583-8555, Japan

**Keywords:** older patients, empowerment, covariance structure analysis, functioning and disability, physical activity, rehabilitation

## Abstract

In the present study, 151 Japanese older adults aged over 65 years and admitted to recovery-phase rehabilitation facilities were enrolled to investigate the relationship between empowerment and contextual factors, functioning and disability, with structural equation modeling (SEM). The analysis included 151 patients aged 81.75 ± 7.15 years, including 54 males (35.76%) and 97 females (64.24%). The results of the SEM analysis showed that role presence (β = 0.45, *p* < 0.01) and family structure (β = 0.18, *p* = 0.02) significantly impacted empowerment. In addition, the results showed that patient empowerment positively impacted physical activity (β = 0.25, *p* < 0.01) and psychosomatic functions and abilities (β = 0.36, *p* < 0.01). Furthermore, the goodness-of-fit of the model hypothesized in this study was shown to have explanatory power. This study showed that empowerment contributed to the prevention of physical inactivity and confinement among Japanese older patients. In other words, the study provided evidence for the importance of empowerment-based program planning in the practice of person-centered care aimed at promoting the health and discharge of older patients in Japan.

## 1. Introduction

Older adults experience changes in self-identity and relationships owing to the onset of diseases, including chronic obstructive pulmonary disease and dementia, leading to powerlessness [1,2]. In particular, older adults who have experienced hospitalization or institutionalization are at a high risk for inactivity owing to their dependence on physicians and allied healthcare professionals for decision making on treatment plans and daily living activities, and they eventually develop learned helplessness [1,2,3]. Therefore, the promotion of individual empowerment in hospitalized older patients, i.e., increased self-awareness of their potential, adaptive behavioral changes towards the maintenance and promotion of health, and retention of the ability to choose various medical and health and welfare services, are necessary to prevent inactivity [4]. In recent years, the paternalistic model of the Japanese healthcare system for older adults has also gradually shifted to a shared decision-making model, which is centered on the advancing empowerment concept [5]. In this context, the concept of empowerment has become particularly important in the care of older Japanese patients.

Empowerment is defined as “the process of helping people assert control over the factors that affect their lives,” and empowerment scales have been developed for older adults [6,7]. In addition, the factors related to empowerment have been progressively revealed, and previous studies have reported their relevance, such as regarding (1) health status (underlying diseases, blood sugar levels, and nutritional status); (2) individual characteristics (age, sex, education level, employment status, quality of life, and subjective well-being); and (3) environmental factors (family structure, quality of care, and social support) [6,8,9]. Other studies have shown that the empowerment of hospitalized patients enhances the two-way communication between patients and healthcare professionals and has a positive impact on functioning and disability, such as improved self-management and health behaviors, self-care and preparation for discharge, and social participation [10,11]. However, these findings are mostly based on young patients and those with specific diseases, and the characteristics of older patients with overlapping diseases and age-related changes, in addition to the physical disease that led to hospitalization, have not yet been clarified. In addition, most researchers reporting on empowerment have comprehensively examined antecedents (factors that influence the occurrence of empowerment) and consequences (factors that are influenced by empowerment) and insufficiently examined the matter along the empowerment concept structure. These may have been influenced by the complexity of the empowerment concept. Moreover, empowerment is strongly influenced by historical backgrounds and cultural differences [12]; therefore, the cultural uniqueness of older adults, including those from Japan, needs to be taken into account, but research and the interpretation of results remain insufficient.

In countries with an increasingly aging population, establishing support methods to prevent learned helplessness and inactivity in older patients immediately after hospitalization and to aid in their early reintegration into community life is needed. Therefore, examining contextual factors that influence empowerment in older Japanese patients and clarifying the relationship and structure between empowerment and functioning and disability may help establish a rationale for designing programs to prevent functional decline and inactivity in older patients in other countries.

The above suggest that the following questions should be asked regarding older patients in Japan: “What influences the occurrence of empowerment?” and “How does empowerment affect the life functions and physical activities of older patients?” Answering these questions objectively and using clinical data, following the structure of the empowerment concept, is necessitated. Therefore, this study clarified the structure of empowerment using the Patient Empowerment Scale—Japanese version (PES-J) and factors that have been shown to be associated with empowerment.

## 2. Materials and Methods

### 2.1. Participants

The inclusion and exclusion criteria used for the selection of participants were as follows:

#### 2.1.1. Inclusion Criteria

(1) The patients or their families consented to participate in this study at the time of admission.
(2) The patients were able to understand the questions (a Mini-Mental State Examination [MMSE] score of 11 or above [13]).

#### 2.1.2. Exclusion Criteria

(1) The patients had a risk of deterioration of their medical condition owing to participation in the survey, as determined by the attending physician.
(2) The patients were unable to understand the questions owing to a decreased level of consciousness or mental function.

The study included patients aged ≥65 years who were admitted to convalescent rehabilitation wards in four Japanese cities (Gose City, Akashi City, Hashimoto City, and Iwata City) between September 2019 and August 2021. These patients were actively rehabilitated for up to 3 h a day for a maximum of 180 days after the onset of illness to facilitate their early discharge. Patients eligible for hospitalization were those who were admitted within 60 days after the onset of different diseases, including central nervous system, orthopedic, and internal disorders [14].

In the sampling procedure, the attending physician first checked for instability of the patient’s medical condition (e.g., abnormal vital signs, such as fever and tachycardia, and disturbances in consciousness, such as delirium) during the first 3 days of hospitalization. Next, the attending physician and occupational therapist explained the purpose and content of the study to all older patients who were deemed eligible for this study and their families, and then followed the procedure to obtain their consent.

In the process of obtaining consent, the participants were provided verbal and written explanations of the study and their participation; their freedom to withdraw from the study at any time with no disadvantages, such as cessation of medical treatment, was assured; and complete protection of their personal information was guaranteed. Subsequently, written informed consent was obtained from the participants.

The target sample size for this study was calculated according to the common guideline for obtaining a trustworthy maximum likelihood (at least 5 cases per model parameter) and using the A-priori Sample Size Calculator for Structural Equation Models (effect size: 0.1, statistical power: 0.8), and required at least 145 cases in the structural equation model analysis.

### 2.2. Survey Items

#### 2.2.1. Selection of Survey Items

Four occupational therapists, one nurse, and one physician with expertise in geriatric psychiatry, all with more than 10 years of work experience in geriatric medicine, selected the survey items according to the following criteria.

#### 2.2.2. Inclusion Criteria

(1) Previous studies have shown the association with empowerment.
(2) The surveys, measurement, and collection of information from the medical records of older patients in the hospital environment and daily care situations were possible.
(3) Those considered necessary for the promotion of health and care of older patients.

#### 2.2.3. Exclusion Criteria

(1) The evaluation/measurement placed a physical and emotional burden on the
(2) Older patient.
(3) The evaluation/measurement may have placed the patient at risk for disease exacerbation.
(4) There was an excessive financial burden for conducting the survey.

#### 2.2.4. Collection of Items

The data from the medical records of the physicians, nurses, and occupational therapists were collected.

#### 2.2.5. Medical Information

The diseases that led to hospitalization, comorbidities, and nutritional status (serum albumin level; Alb) were investigated, and the Charlson Comorbidity Index (CCI), which predicts the short-term risk of death in patients with comorbidities, was calculated [15].

#### 2.2.6. Individual Patient Characteristics

The patient’s age, sex, years of education, family structure, relationship with the primary caregiver, economic status, and hobbies/roles were surveyed. The qualitative survey items were categorized and recorded according to 4-point categorical variables: family structure (1 = lone parent, 2 = married couple, 3 = two-parent household, and 4 = three or more-person household) and primary caregiver (1 = spouse of the child, 2 = daughter/son, 3 = sibling, and 4 = spouse); 3-point categorical variables: economic status (1 = independent, 2 = family support, and 3 = public support); and 2-point binary variables for hobbies and roles (1 = yes and 2 = no). In addition, the questions for each variable were: for family structure “Who are the family members with you?”; primary caregiver, “Do you have a primary caregiver?”; economic status, “Are you currently receiving economic support?”; and hobbies and roles, “Do you have a hobby/role in the community or at home?”.

#### 2.2.7. Evaluation and Measurement Items

The results of the individual evaluation conducted by the occupational therapist in charge were adopted. The evaluators had experience in evaluating all survey items. In addition, the evaluations were conducted within one week of admission.

#### 2.2.8. Empowerment

The PES-J [16], a patient-based scale that is used to evaluate the frequency of daily care and environmental adjustments that are related to empowerment using a 4-point categorical scale (Never/Sometimes/Often/Always), was used. The PES-J uses empowerment, defined as “the series of processes in which disclosing oneself, not only verbally but also nonverbally (e.g., through work, roles, and collaborative activities), in connection with others, objectively perceiving one’s existence and challenges, taking proactive actions based on decision-making, and utilizing one’s strengths in new work and community life.”

The PES-J consists of 37 items, 20 of which are care-related questions that have a positive impact on empowerment (e.g., “Do staff make sure that you can reach a nurse call?”), and the other 17 questions have a negative impact on empowerment (e.g., “Do staff move your bed and locker in your room in a manner that is against your preference?”). The PES-J has a 6-factor structure, consisting of subject–staff interaction, environmental adjustment through collaboration, the gathering of the necessary information and problem awareness, proactive behavioral practices, self-disclosure, and self-management of activities, based on confirmatory factor analysis. The model fit for the 6-factor model was indicated by the comparative fit index (CFI) = 0.89, goodness-of-fit index (GFI) = 0.81, adjusted goodness-of-fit index (AGFI) = 0.75, and root mean square error of approximation (RMSEA) = 0.08. Cronbach’s alpha was 0.93 for all 37 items. Cronbach’s alphas for the six factors were, in order, 0.93, 0.91, 0.91, 0.92, 0.91, and 0.75, respectively. In the PES-J scoring, scores for positive questions were added (0 to 3) and negative questions were subtracted (−3 to 0), with the total scores ranging from −51 to 60; lower total scores indicated a state of learned helplessness.

#### 2.2.9. Cognitive Function

The MMSE, which is used to assess general cognitive function on a 30-point scale, was implemented [17]. The MMSE consists of three factors and 11 items, including “simple memory tasks,” “disorientation tasks”, and “tasks requiring spontaneous thinking” for older adults, and a lower score indicates poorer cognitive function [18]. The Japanese version of the MMSE, with previously shown validity and reliability in older adults, has an internal consistency of 0.73 or more for Cronbach’s alpha [18,19]. In Japan, the MMSE is used as a screening tool for dementia, and a score of fewer than 24 points indicates suspected dementia.

#### 2.2.10. Muscle Strength

The strength of the right and left quadriceps muscles was measured using manual muscle testing (MMT), which includes grading on a 6-point categorical scale from 0 (no muscle contraction) to 5 (normal) [20]. MMT is used globally and has demonstrated clinical utility for patients of various ages and diseases.

#### 2.2.11. Pain

The Numerical Rating Scale (NRS), an 11-point categorical scale from 0 (no pain) to 10 (worst possible pain), was used [21]. It consisted of 1 question and was rated by asking, “Taking the strongest pain you have ever experienced as 10, how is your current pain?” Judgments were expressed on a scale of 1 to 3 for mild, 4 to 6 for moderate, and 7 to 10 for severe, and the scale was indicated for application to older patients.

#### 2.2.12. Activities of Daily Living

Activities of daily living (ADL) consisted of 13 motor and 5 cognitive items, and the functional independence measure (FIM) was used to evaluate each item on a 7-point categorical scale from 7 (complete independence) to 1 (total assistance) (total: 18–126 points) [22]. The FIM is considered an important factor influencing the discharge of older patients to home and is associated with several factors, including quality of life and care burden. It has also been shown to be clinically useful in older patients with stroke, orthopedic diseases, and medical conditions.

#### 2.2.13. Physical Activity

A uniaxial accelerometer (LC) (LifeLyzer05 Coach, Suzuken Corporation, Nagoya, Japan) was used to measure low-intensity (1–3 metabolic equivalents [METs]; low activity), medium-intensity (4–6 METs; medium activity), and high-intensity (7–9 METs; high activity) activity time [23]. Instructions, including precautions, on wearing and using the LC device were given to the occupational therapist in charge and to the patients verbally and in writing. In addition, the patients were instructed to wear the device throughout the day for 7 days, except during bathing and sleeping [24]. In the final analysis, the data from the patients who were able to wear the LC and record their activities were used to calculate the mean MET values for the 7 days.

### 2.3. Data Analytical Strategy

**Figure 1:**
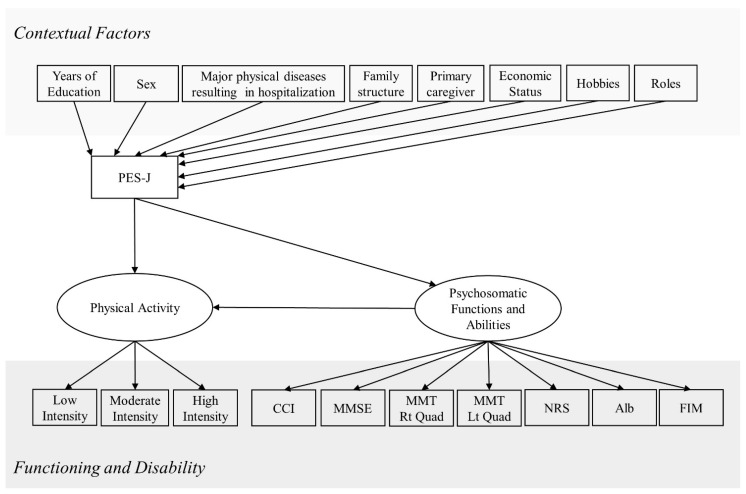
Empowerment structure model based on the hypothesis. CCI: Charlson Comorbidity Index, MMSE: Mini-Mental State Examination, MMT: manual muscle testing, NRS: Numerical Rating Scale, Alb: serum albumin test, FIM: functional independence measure, PES-J: Patient Empowerment Scale—Japanese Version.

First, the survey items were classified into “Contextual Factors” and “Functioning and Disabilities” based on the International Classification of Functioning, Disability and Health. Four occupational therapists (Y.T., A.T., K.S., and K.Y.) and a physician (M.K.) discussed and developed the empowerment structure model based on the hypothesis that “empowerment is generated and promoted by the influence of contextual factors and affects functioning and disabilities” (Figure 1). The empowerment structure model classified functioning and disabilities into two latent variables according to survey item characteristics and named each latent variable “Physical activity” and “Psychosomatic Functions and abilities”.

Next, for verifying univariate normality, skewness and kurtosis were calculated, and survey items that did not meet the safe cutoff criteria of skewness, <2 in absolute value, and kurtosis, <7 in absolute value, were excluded from the analysis [25]. Furthermore, a single regression analysis was conducted on the PES-J and survey items for the purpose of selecting the observed items to be included in the structural model. For contextual factors that are predicted to have an impact on empowerment, the analysis was conducted with the PES-J as the dependent variable and contextual factors as the independent variables. For functioning and disability, which are predicted to have an impact on empowerment, analyses were conducted with each factor categorized as functioning, using disability as the dependent variable and the PES-J as the independent variable. Based on the results of both analyses, items with *p* > 0.05 were excluded from the analysis after careful discussion of their necessity for hypothesis testing. Spearman’s rank correlation coefficients were calculated for the observed items included in the analysis, and multicollinearity was determined to be present when ρ > 0.90.

Once the observed variables involved in the empowerment structure model were finalized, multivariate normality was verified using Mardia’s two-sided test of fit for skewness and kurtosis. For the two latent variables included in the empowerment structure model, the tau-equivalence assumption was first tested for internal consistency, with Cronbach’s alpha calculated for success (*p* ≥ 0.05) and McDonald’s omega for failure (*p* < 0.05). If there were fewer than three observed items in the latent variable, Cronbach’s alpha was selected. Similarly, convergent and discriminant validities were verified using the values of composite reliability (CR) and average variance extracted (AVE). The respective criteria were Cronbach’s alpha > 0.07 and McDonald’s omega > 0.80 for internal consistency, and AVE > 0.50, CR > 0.60 for convergent validity [26,27]. The criterion for discriminant validity was that the square root of AVE was greater than the inter-factor correlations [27]. In addition, a confirmatory factor analysis was conducted to validate the factor structure, and χ2, GFI, AGFI, CFI, and RMSEA were calculated [28,29]. Regarding the adoption criteria for each value, χ^2^ was judged to have *p* > 0.05; GFI, AGFI, and CFI to have >0.90; and RMSEA to have <0.05, indicating a “fit” model.

After confirming the validity of the factor structure, the empowerment structure model was analyzed using structural equation modeling (SEM). In the SEM, the correlation (ρ) and standardized partial regression (β) coefficients between the items and the square of the multiple correlation coefficient (R^2^) of the items were calculated. The goodness-of-fit of the model was performed by calculating the degrees of freedom and χ^2^ values, GFI, AGFI, CFI, and RMSEA to present a multidimensional perspective. The model with the lowest Akaike Information Criterion (AIC) was adopted for relative model validation. The statistical analysis of the data was performed using IBM SPSS Statistics Ver. 28.0, IBM SPSS Amos Ver. 28.0 (IBM Corp., Armonk, NY, USA) and R Ver. 4.0.5 (R Foundation for Statistical Computing, Vienna, Austria), with a significance level of ˂5%.

## 3. Results

### 3.1. Participant Characteristics

Out of the 159 patients who consented to participate in the study, two withdrew owing to the deterioration of their condition, and six had difficulty in recording physical activity due to an inability to continuously wear the device during the survey. The characteristics of the final 151 participants and the results of the entire survey are presented in Table 1. There were 54 males (35.76%) and 97 females (64.24%), and the mean age was 81.75 ± 7.15 years; 33, 79, and 39 patients were hospitalized for central nervous system disorders, orthopedic diseases, and disuse syndrome associated with medical diseases, respectively.

### 3.2. Process for Selecting the Items Constituting the Structural Model

There were no missing values because the study population consisted of patients at a low risk for health exacerbations during hospitalization, and the investigation was conducted by full staff review. In the univariate normality test, the skewness was 4.08 and kurtosis 23.33 for high-intensity activity time, which was excluded because it did not meet the set cutoff criteria. The single regression analysis showed that among the contextual factors, family structure (β = −0.18; *p* = 0.02) and roles (β = −0.41; *p* < 0.01) were significant influence items for empowerment (Table 2). The PES-J was also found to significantly influence CCI (β = −0.29; *p* < 0.01); MMSE (β = 0.22; *p* < 0.01); NRS (β = −0.17; *p* = 0.04); Alb (β = 0.30; *p* < 0.01); FIM (β = 0.32; *p* < 0.01); low-intensity activity time (β = 0.45; *p* < 0.01); and moderate-intensity activity time (β = 0.38; *p* < 0.01) among the functioning and disability factors (Table 3). Finally, the clinical significance of the above items and their application to SEM were examined. No multicollinearity by correlation coefficients was found among the observed variables used in the SEM (Table 4).

### 3.3. Relationship between Factor Structure in Functioning and Disability and Empowerment

**Figure 2:**
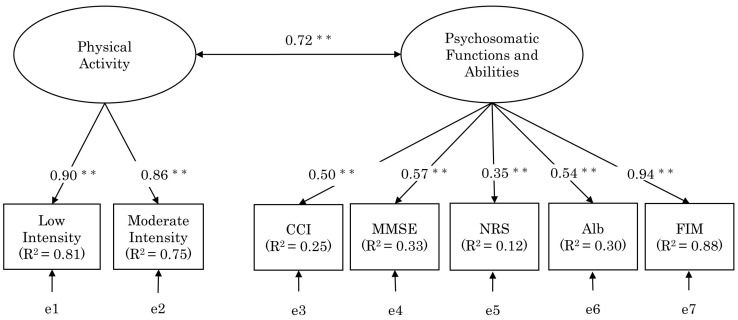
Confirmatory factor analysis of functioning and disabilities. CCI: Charlson Comorbidity Index, MMSE: Mini-Mental State Examination, NRS: Numerical Rating Scale, Alb: serum albumin test, FIM: functional independence measure, PES-J: Patient Empowerment Scale—Japanese Version. **: *p* < 0.01.

The test of multivariate normality showed a multivariate skewness of 412.48 (*p* < 0.01) and a multivariate kurtosis of 1.51 (*p* = 0.13). As the multivariate normality test showed that the data did not follow a normal distribution, the analyses were validated with diagonally weighted least squares [25]. The constructs of functioning and disability were two latent factors and seven observed factors; the two latent factors consisted of physical activity with low- and moderate-intensity activity time as the observed factors, and psychosomatic functions and abilities with CCI, MMSE, NRS, Alb, and FIM as the observed factors (Figure 2). The CCI and NRS were reverse-scored and used in the analysis, as higher scores indicate worse status. The result of the tau-equivalence assumption test for the observed variables was *p* < 0.01. Therefore, for internal consistency, Cronbach’s alpha was calculated for physical activity, which consisted of two observed variables, and McDonald’s omega for psychosomatic functions and abilities, resulting in Cronbach’s alpha with 0.73 and McDonald’s omega with 0.92. The convergent validity showed that CR = 0.98 and AVE = 0.77 for physical activity, and CR = 0.73 and AVE = 0.37 for psychosomatic functions and abilities, with the AVE for psychosomatic functions and abilities falling below the criterion. Furthermore, for discriminant validity, the square root of the AVE was 0.88 for physical activity and 0.61 for psychosomatic functions and abilities, and the AVE for psychosomatic functions and abilities was below the inter-factor correlation. The model’s goodness-of-fit revealed a statistically acceptable factor structure with 13 degrees of freedom, χ^2^ value = 5.06 (*p* = 0.97), GFI = 0.99, AGFI = 0.98, CFI = 1.00, AIC = 35.06, and RMSEA < 0.01.

**Figure 3:**
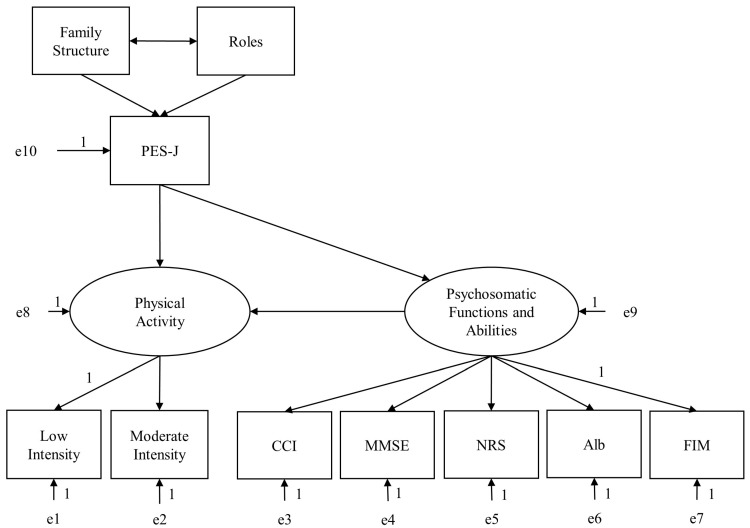
Form of the causal hypothesis model used in the analysis. PES-J: Patient Empowerment Scale—Japanese Version, CCI: Charlson Comorbidity Index, MMSE: Mini-Mental State Examination, NRS: Numerical Rating Scale, Alb: serum albumin test, FIM: functional independence measure.

**Figure 4:**
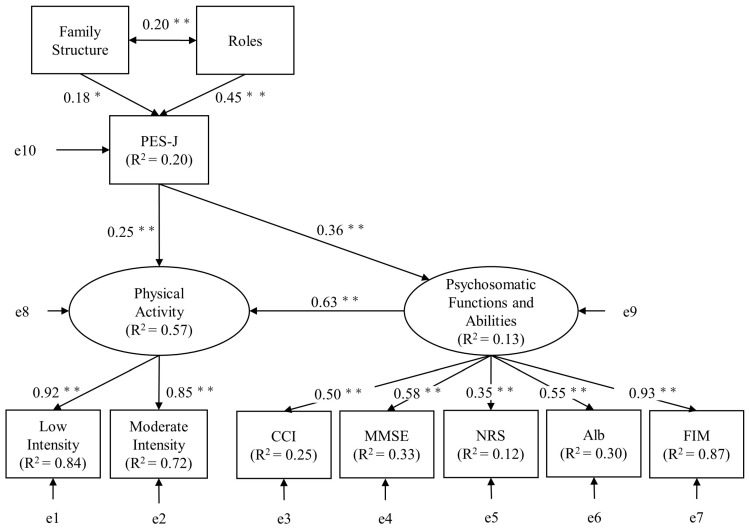
Standardized estimates of the structural equation modeling. PES-J: Patient Empowerment Scale—Japanese Version, CCI: Charlson Comorbidity Index, MMSE: Mini-Mental State Examination, NRS: Numerical Rating Scale, Alb: serum albumin test, FIM: functional independence measure, *: *p* < 0.05, **: *p* < 0.01.

Next, in the analysis of the empowerment structure model, one of the path coefficients of the latent variables of physical activity and psychosomatic functions and abilities to the observed variables was each constrained to 1. In addition, the endogenous variables of “physical activity” and “psychosomatic functions and abilities” were associated with a disturbance variable, and each observed variable, which was the dependent variable, was associated with an error variable; all path coefficients for the disturbance and error variables were fixed at 1 (Figure 3). In addition, the roles were reversed to “1 = unaffected, 2 = affected” for the analysis. The results of the SEM are presented in Figure 4. In terms of factors affecting empowerment, the path coefficient of roles to PES-J was β = 0.45 (*p* < 0.01) and from family structure to PES-J was β = 0.18 (*p* = 0.02). In the impact of empowerment, the path coefficient of PES-J to physical activity was β = 0.25 (*p* < 0.01); PES-J to psychosomatic functions and abilities was β = 0.36 (*p* < 0.01); and psychosomatic functions and abilities to physical activity was β = 0.63 (*p* < 0.01). The mediated effect and standardized mediated effect from PES-J to physical activity were 0.16 and 0.23, respectively. Activity time according to the intensity of physical activity showed R^2^ = 0.84 and R^2^ = 0.72 for low and moderate intensities, respectively. Psychosomatic functions and abilities showed R^2^ = 0.25, R^2^ = 0.33, R^2^ = 0.12, R^2^ = 0.30, and R^2^ = 0.87 for CCI, MMSE, NRS, Alb, and FIM, respectively. The model’s goodness-of-fit indicated by 32 degrees of freedom; χ^2^ value = 25.38 (*p* = 0.79); GFI = 0.97, AGFI = 0.95; CFI = 1.00; AIC = 71.38, and RMSEA < 0.01.

## 4. Discussion

We examined the relationship between the empowerment of older patients and contextual factors, functioning and disability using SEM. The results revealed that the empowerment of older patients in the early post-hospitalization period was influenced by their roles and family structure, which in turn, affected their physical activity, psychosomatic functions and abilities. The results supported the findings of previous review studies that were performed to qualitatively examine the empowerment of older adults in Japan [8]. Furthermore, the empowerment structure model created in this study suggested that the structure is statistically acceptable, as indicated by the model’s goodness-of-fit.

### 4.1. Contextual Factors Affecting Empowerment

Of the contextual factors, roles were shown to have the greatest impact (β = 0.45; *p* < 0.01) on empowerment. The question “Do you have a role in the community or at home?” was used to assess patients’ subjective role perceptions. In an interview study of community-dwelling older adults in Japan, the desire to hold on to roles for others was shown to lead to empowerment [30]. The study also reported that role recognition leads to a greater range of activities and less loneliness and motivates these older adults to engage in new activities. Although the participants of this study were inpatients, the perception and expectation that they have a role to play were considered to be important factors in empowerment, as in previous studies. Furthermore, empowerment comprised the following components: (1) internal (the individual’s ethos); (2) interactional (interaction with the environment surrounding the individual); and (3) behavioral (actions that involve and build functional relationships with local groups and organizations) [12]. The results of this study indicated that in older patients, the perception of having a role, either during hospitalization or after discharge, may have influenced empowerment by providing physical (interactional) and functional (behavioral) connections within their homes and the society. Furthermore, verbal and non-verbal communication with others through roles and activities was demonstrated to be useful for the empowerment of older Japanese adults. Role recognition is the perception that one can make a difference via non-verbal interaction with others and the surrounding environment, and the results of this study indicated that role recognition is effective in empowering older patients and, consequently, in controlling learned helplessness.

Next, family structure was also shown to be a contextual factor affecting empowerment (β = 0.18; *p* = 0.02) and correlated with roles (ρ = 0.20; *p* < 0.01). In Japanese traditional culture, older adults live with their children and grandchildren, and family and neighbors have considered it important for them to feel safety and relief by supporting each other [31]. Based on these cultural backgrounds, cohabitation with family was considered to be a contributing factor to the positive impact on empowerment by introducing trust and security to older patients. Furthermore, the correlation between family structure and roles suggested that the perception of roles within the family contributes to the occurrence of empowerment. In particular, according to the result of this study, cohabitation with family who were generations apart had more influence on empowerment and was considered to be one of the characteristics among older patients in Japan. In recent years, social distancing due to COVID-19 has resulted in loneliness and social isolation among older adults, thereby leading to anxiety, depression, poor sleep quality, and physical inactivity [32]. In these situations, the presence of a family member who enabled daily conversations also shared activities, and role recognition was considered an important factor influencing the empowerment of older patients. Physical, mental, and physiologic decline in older adults decreases their tolerance for environmental changes and increases their risk of feeling powerless [9,33,34]. Additionally, self-efficacy and self-esteem, the constructs of the PES-J, have been reported to decrease owing to irreversible age-related changes (health, life, and environment) [35]. Therefore, based on the results of this study, it was thought that empowerment-based support and environmental adjustments would be needed more in older than in younger patients.

### 4.2. Impact of Empowerment on Functioning and Disability

Zimmerman reported that empowerment affects the initiative and motivation to participate in society [12]. Consistent with the findings of previous studies, the results of the present study indicate that empowerment significantly affects physical activity in older patients (β = 0.25; *p* < 0.01). Furthermore, this study used activity time based on intensity as a measure of physical activity and found that low-intensity activity time showed the strongest coefficient of determination. Low-intensity activity is a static activity performed in a sitting or standing position that has little influence on ADL independence and physical and mental functions [36]. Disease prevention and health improvement require appropriate modification of physical activity intensity according to the individual’s exercise experience and physical ability, and recent studies have suggested that increased low-intensity activity time is effective in preventing dementia and maintaining and improving quality of life [34,37,38]. Older patients in the early stages of hospitalization often have limited activities due to a temporary or chronic decline in physical and mental functions caused by illness or age-related changes; therefore, empowerment promotes low-intensity activities in the sitting and standing positions and is an important factor in the prevention of inactivity.

Furthermore, this study showed that empowerment had a significant effect on psychosomatic functions and abilities (β = 0.36; *p* < 0.01). In addition, the FIM showed the largest coefficient of determination as a measure of psychosomatic functions and abilities. The FIM determines ADL independence as the amount of assistance needed by caregivers and represents the degree of patient dependence on caregivers, such as in relation to the sense of burden of caregiving. Previous studies have shown that empowerment has a strong influence on the self-management of life, health (e.g., blood glucose levels, mental status), and activities [6,9,10]. Similarly, in older patients in Japan, the present results suggested that empowerment might influence the self-management of ADL and independent consciousness. In addition, empowerment was found to affect systemic conditions, such as comorbidities and nutritional status, as well as psychosomatic functions, such as cognitive function and pain. The empowerment of older adults involves a series of processes based on decision making, wherein self-awareness of health status and challenges leads to proactive behavioral self-management [8]. As psychosomatic functions and abilities showed an impact on physical activity in this study, improvement in ADL, comorbidities, nutrition, pain, and cognitive function is the main factor leading to proactive physical activity. In other words, it was inferred that the improvement in psychosomatic functions and abilities due to the empowerment of older patients may indirectly contribute to increased physical activity.

The hypothesized empowerment structure model proposed in the present study revealed a good statistical fit. These results indicate that the empowerment of older patients might have a positive impact on overall life functioning through activity choice and the decision to practice and might be effective in preventing confinement and frailty.

### 4.3. The Application of Empowerment in Older Adults’ Healthcare

The concept of empowerment in healthcare includes the process of adaptation from the patient’s own choice of treatment, goals, and environment, and the recognition of abilities and challenges in the patient–healthcare professional interaction through conversation and work activities [8]. Appropriate voice and environmental adjustments in practical care situations that consider the older patient’s individuality have been reported to fulfill their potential abilities, and this realization of functional improvement influences the expectation and practice of new activities [8]. The results of the present study reiterated the importance of empowerment-conscious medical and healthcare practices in older patients who present with learned helplessness and are at a high risk for becoming bedridden [7,33]. Furthermore, being bedridden is a significant factor that impedes the recovery of function and complicates the achievement of the desired outcomes for the patients and their families [39]. Therefore, healthcare professionals need to provide support aimed at preventing learned helplessness by sufficiently communicating with the patients, adjusting the environment according to their individuality in daily care situations, and sharing and practicing the treatment, goals, and life chosen by the patients.

Stichler reported that empowerment promotes a patient’s readiness for discharge (activation) [11]. Preparation for discharge is not only the adjustment of the physical environment for discharge, such as the living environment and welfare equipment, but also the acquisition of knowledge and skills for the self-management of activities and social participation, in order to regain community life in accordance with the person’s wishes and values. Furthermore, roles at home and social activities are useful in maintaining and improving the amount of physical activity in older adults [40]. Medical institutions that support the discharge of older patients to the community need to intervene with an eye towards role fulfillment and social participation in post-discharge life. To this end, it is important to strengthen person-centered care more than ever; to share the patient’s goals and challenges with family members and medical professionals; to acquire knowledge and skills for social participation and to strengthen their effectiveness; and to actively implement programs to resume social participation according to the individual’s will. Therefore, given the results of this study, hospitalization support with an awareness of empowerment would prevent physical inactivity in older patients, promote discharge to the familiar environment where these patients wish to reside, and lead to a smooth resumption of community life.

### 4.4. Limitations

First, discriminant validity was insufficient for the two latent variables in functioning and disability. This result may have been influenced by the fact that each of the observed variables in functioning and disability, with the exception of the NRS, were related to each other. Our study was limited to the factors related to empowerment that could be measured in older patients in a clinical setting; hence, the survey items related to life function need to be reexamined and adjusted. Second, the participants in this study were older Japanese patients, and characteristics of generational and cultural differences could not be verified. In addition, generalization may not be possible owing to the limited number of participants. Third, the present study was a cross-sectional survey of older patients in the early post-hospitalization period; hence, the utilization and effects of empowerment on care were unclear. Therefore, it is necessary to improve the model for clinical application by examining changes over time and verifying its validity during the course of the study and at the time of discharge from the hospital. In addition, the survey should be expanded to include a larger area and sample size, as well as consideration of the local environment and cultural characteristics of older adults.

## 5. Conclusions

In the present study, we examined the association between empowerment and contextual factors, functioning and disability among older patients in Japan using clinical data from 151 patients. The results of the SEM analysis confirmed the validity of the research hypotheses that the promotion of empowerment affects the amount of physical activity, as it was found that the empowerment of older patients was influenced by role perception and family structure, which in turn, affected physical activity, psychosomatic functions and abilities. In particular, the strong impact on low-intensity physical activity and ADL was significant. This suggested that low-intensity physical activity was less physically restricting than high-intensity physical activity, and that older patients in the early stages of hospitalization with low levels of independence were able to select and perform activities according to their conditions and abilities. Therefore, the results of this study may help to establish care and support to prevent inactivity and confinement in older patients with physical illnesses and age-related changes. In addition, it may be necessary to develop programs to promote the empowerment of older patients, with the intention of acquiring roles appropriate to their family environment.

## Figures and Tables

**Table 1:** Characteristics of participants and scores for each assessment. (N = 151).

Item	N = 151		Skewness	Kurtosis
Age (years)	81.75	−7.15	−0.35	−0.15
Years of education	11.2	−1.81	0.45	0.5
Sex (no. of people)				
Male	54	35.76%	−0.76	−1.45
Female	97	64.24%		
Major physical diseases resulting in hospitalization (no. of people)				
Central nervous system diseases	33	21.85%		
Orthopedic diseases	79	52.32%	−0.05	−0.89
Disuse syndromes associated with medical diseases	39	25.83%		
CCI	1.56	−1.31	0.83	0.92
MMSE	22.09	−5.31	−0.4	−0.77
MMT				
Right quadriceps	3.79	−0.71	−0.5	1.11
Left quadriceps	3.69	−0.6	0.07	−0.37
NRS	3.48	−2.2	0.31	−0.96
Alb (g/dL)	3.74	−0.39	−0.11	−0.53
FIM	73.22	−20.91	0.06	−0.53
Activity time per intensity (minute)				
Low intensity	13.6	−12.14	1.02	0.19
Moderate intensity	4.31	−5.3	1.9	3.79
High intensity	0.59	−0.99	4.08	23.33
Family structure (no. of people)				
Lone parent	59	39.07%		
Married couple	23	15.23%	0.19	−1.39
Two-parent household	52	34.44%		
Three or more-person household	17	11.26%		
Primary caregiver (no. of people)				
Spouse of child	26	17.22%		
Daughter/Son	96	63.58%	0.95	0.07
Sibling	2	1.32%		
Spouse	27	17.88%		
Economic status (no. of people)				
Independent	84	55.63%		
Family support	58	38.41%	0.79	−0.34
Public support	9	5.96%		
Hobbies (no. of people)				
Affected	95	62.91% 37.09%	0.54	−1.73
Unaffected	56			
Roles (no. of people)				
Affected	85	56.29%	0.26	−1.96
Unaffected	66	43.71%		

CCI: Charlson Comorbidity Index, MMSE: Mini-Mental State Examination, MMT: manual muscle testing, NRS: Numerical Rating Scale, Alb: serum albumin test, FIM: functional independence measure.

**Table 2:** Results of single regression analysis: contextual factors affecting empowerment (N = 151).

Independent Variable	*B*	*SE B*	*β*	*t* Value	*f ^2^*
Years of education	−0.13	0.72	−0.01	−0.17	<0.01
Sex	−3.25	2.77	−0.10	−1.17	<0.01
Major physical diseases resulting in hospitalization	−0.40	1.89	−0.02	−0.21	<0.01
Family structure	2.68	1.16	0.18 *	2.32	<0.01
Primary caregiver	−0.13	1.41	−0.01	−0.09	<0.01
Economic status	−0.79	2.15	−0.03	−0.37	<0.01
Hobbies	−3.23	2.69	−0.10	−1.20	<0.01
Roles	−13.26	2.39	−0.41 **	−5.54	0.20

PES-J: dependent variable, *: *p* < 0.05, **: *p* < 0.01, SE: standard error, PES-J: Patient Empowerment Scale—Japanese Version.

**Table 3:** Results of single regression analysis: empowerment affecting functioning and disability factors (N = 151).

Dependent Variable	*B*	*SE B*	*β*	*t* Value	*f ^2^*
CCI	−0.64	0.18	−0.29 **	−3.64	0.09
MMSE	0.07	0.03	0.22 **	2.75	0.05
MMT					
Right quadriceps	<0.01	<0.01	0.04	0.49	<0.01
Left quadriceps	<0.01	<0.01	0.11	1.30	<0.01
NRS	−0.02	0.01	−0.17 *	−2.04	0.03
Alb	0.01	<0.01	0.30 **	3.89	0.10
FIM	0.41	0.10	0.32 **	4.06	0.11
Activity time per intensity					
Low intensity	0.34	0.06	0.45 **	6.19	0.27
Moderate intensity	0.13	0.03	0.38 **	5.00	0.16

PES-J: independent variable, *: *p* < 0.05, **: *p* < 0.01, SE: Standard error. PES-J: Patient Empowerment Scale—Japanese Version, CCI: Charlson Comorbidity Index, MMSE: Mini-Mental State Examination, MMT: manual muscle testing, NRS: Numerical Rating Scale, Alb: serum albumin test, FIM: functional independence measure.

**Table 4:** Correlation matrix of continuous variables analyzed in the structural equation modeling (N = 151).

	PES-J	CCI	MMSE	NRS	Alb	FIM	Low-Intensity Activity Time	Moderate-Intensity Activity Time
PES-J	1							
CCI	−0.25 **	1						
MMSE	0.20 *	−0.20 **	1					
NRS	−0.16 *	0.11	−0.19 *	1				
Alb	0.32 **	−0.23 **	0.31 **	−0.25 **	1			
FIM	0.34 **	−0.38 **	0.53 **	−0.36 **	0.54 **	1		
Low-intensity activity time	0.45 **	−0.27 **	0.40 **	−0.27 **	0.34 **	0.63 **	1	
Moderate-intensity activity time	0.35 **	−0.26 **	0.44 **	−0.30 **	0.30 **	0.64 **	0.85 **	1

*: *p* < 0.05, **: *p* < 0.01. PES-J: Patient Empowerment Scale—Japanese Version, CCI: Charlson Comorbidity Index, MMSE: Mini-Mental State Examination, NRS: Numerical Rating Scale, Alb: serum albumin test, FIM: functional independence measure.

## Data Availability

Owing to privacy and ethical concerns, neither the data nor the data source can be made available.
